# A holistic approach to preventing type 2 diabetes in Asian women with a history of gestational diabetes mellitus: a feasibility study and pilot randomized controlled trial

**DOI:** 10.3389/fcdhc.2023.1251411

**Published:** 2023-09-28

**Authors:** Seaw Jia Liew, Chun Siong Soon, Yu Chung Chooi, Mya Thway Tint, Johan Gunnar Eriksson

**Affiliations:** ^1^ Human Potential Translational Research Program, Yong Loo Lin School of Medicine, National University of Singapore, Singapore, Singapore; ^2^ Centre for Sleep and Cognition, Yong Loo Lin School of Medicine, National University of Singapore, Singapore, Singapore; ^3^ Singapore Institute for Clinical Sciences, Agency for Science, Technology and Research (A*STAR), Singapore, Singapore; ^4^ Department of Obstetrics and Gynecology, Yong Loo Lin School of Medicine, National University of Singapore, Singapore, Singapore; ^5^ Folkhälsan Research Center, Helsinki, Finland

**Keywords:** diabetes, gestational diabetes mellitus, feasibility, pilot, randomized controlled trial, holistic, Asian, women

## Abstract

**Background:**

Gestational Diabetes Mellitus (GDM) exposes women to future risk of Type 2 Diabetes. Previous studies focused on diet and physical activity, less emphasis was given to tackle intertwined risk factors such as sleep and stress. Knowledge remains scarce in multi-ethnic Asian communities. This study explored the: (1) feasibility of a holistic digital intervention on improving diet, physical activity (PA), sleep and stress of Asian women with a history of GDM, and (2) preliminary efficacy of the holistic intervention on women’s physical and mental well-being via a pilot randomized controlled trial.

**Methods:**

Female volunteers with a history of GDM but without pre-existing diabetes were recruited from multi-ethnic Singaporean community. Each eligible woman was given a self-monitoring opportunity using Oura Ring that provided daily feedback on step counts, PA, sleep and bedtime heart rate. Intervention group additionally received personalized recommendations aimed to reinforce healthy behaviors holistically (diet, PA, sleep and stress). Dietary intake was evaluated by a research dietitian, while step counts, PA, sleep and bedtime heart rate were evaluated by health coaches based on Oura Ring data. Perceived physical and mental health and well-being were self-reported. Clinical outcomes included glycemic status determined by HbA_1c_ and OGTT tests, body mass index, blood pressures and lipid profile.

**Results:**

Of 196 women from the community, 72 women completed diabetes screening, 61 women were eligible and 56 women completed the study. The 56 completers had mean age of 35.8 ± 3.7 years, predominantly Chinese, majority had their first GDM diagnosed at least 2 years ago and had two GDM-affected pregnancies. After intervention period, more women in the Intervention group achieved at least 8,000 steps/day and had at least 6 hours of sleep per night. Noticeable reduction of added sugar in their food and beverages were observed after the dietary intervention. Changes in body weight and mental well-being were observed but group differences were not statistically significant.

**Conclusions:**

The holistic approach appeared feasible for personalizing lifestyle recommendations to promote physical and mental well-being among women with a history of GDM. Larger studies with sufficient assessment timepoints and follow-up duration are warranted to improve the evaluation of intervention effects on clinical outcomes.

**Clinical trial registration number:**

https://clinicaltrials.gov/show/NCT05512871, NCT05512871.

## 1 Introduction

Women with a history of Gestational Diabetes Mellitus (GDM) have an increased risk for Type 2 Diabetes (T2D) and other noncommunicable diseases than women who had normoglycemic pregnancy (1 ; 2–5). On a global scale, the prevalence of T2D including GDM was estimated to change at different rates in different regions (6). The age-adjusted hyperglycemia in pregnancy rate was projected to be around 15.8-16.0% between 2019 and 2045 contingent on other characteristics and the use of diagnostic criteria specific to the regions (7).

GDM is defined as glucose intolerance with first onset during pregnancy. While GDM may resolve following pregnancy, compared to women who had a normoglycemic pregnancy, women with a history of GDM are at a 10-fold risk of developing T2D (8). Risk factors for GDM are largely similar to those for T2D (e.g. family history of T2D, ethnicity, advanced age and overweight or obesity) (9). Pregnancy related risk factors (e.g. increased age in pregnancy, insulin therapy, multiparity, hypertensive disorders and preterm delivery) and risk factors after pregnancy (e.g. weight gain and physical inactivity) were also associated with future development of T2D (4, 9). The risk of T2D is elevated with the increasing length of GDM history. Accordingly, women with recent GDM could benefit from early intervention to help them prevent T2D or delay the progression to T2D (10–12).

Mobile health (mHealth) and digital interventions have emerged and played a transformative role in facilitating lifestyle changes by supplementing face-to-face interventions and bringing convenience to the users. A multitude of smartphone Applications (App), wireless mobile technologies, virtual communication tools and activity tracking wearable devices have been introduced to the space of health promotion, care delivery, disease prevention and management (13). While mHealth is beginning to gain traction among relevant stakeholders in the healthcare industry, it is of our interest to explore the possibility of leveraging a mHealth strategy to address the health needs of women with a history of GDM in a holistic manner.

Previous prevention studies have largely been focusing on promoting healthy diet and physical activity and less emphasis was given to tackle the intertwined risk factors for T2D such as sleep and stress. Furthermore, evidence of lifestyle intervention on preventing T2D in women with a history of GDM remains scarce in the context of multi-ethnic Asian community and this calls for further exploration. Therefore, this study aimed to: 1a) examine the feasibility of using smartphone Apps and activity wearable device to collect data (diet, PA, sleep and stress) necessary for a multi-disciplinary team of health coaches to deliver holistic lifestyle interventions that can be personalized and tailored to the needs of women at an individual level; 1b) explore whether the lifestyle data collected within a short time window (10 weeks) can provide meaningful information for understanding lifestyle behavior in a holistic manner and facilitate health coaching, and 2) explore preliminary efficacy of the proposed lifestyle intervention on clinical health and well-being outcomes (blood glucose concentrations, body weight, blood pressures, lipid profile, mental well-being and perceived general well-being) among women with a history of GDM via a pilot randomized controlled trial (RCT) study design.

## 2 Materials and methods

### 2.1 Study characteristics

#### 2.1.1 Study design

This was a feasibility and pilot study conducted among Asian women with a history of GDM. The feasibility study lasted for 10 weeks in total. The study design included a 2-week baseline observation phase followed by a 8-week pilot study that comprised a 4-week RCT (parallel two-arm, single-center, non-blinded) and a 4-week follow-up period.

#### 2.1.2 Eligibility criteria

Inclusion criteria were Asian women residing in Singapore during the study period, aged between 21-45 years who self-reported a history of GDM of not more than 10 years ago, without pre-existing diabetes, and able to provide a written informed consent in English language. Exclusion criteria were current or previous diagnosis of diabetes mellitus (except GDM), currently pregnant or anticipate becoming pregnant, performing full breastfeeding during study period, within 12 weeks postpartum, following special diet or dietary restrictions, experiencing severely limited mobility, diagnosed with malnutrition, eating disorders, severe insomnia or major diseases (cancers, unstable heart diseases, severe kidney diseases, unstable mental illness, dementia or cognitive impairment), experiencing alcohol or drug abuse, taking medications known to influence glucose metabolism, did not own a smartphone (Android version 10 or above) compatible with the mobile Apps used in this study, and participating in concurrent clinical trial, lifestyle intervention or fitness and wellness program.

#### 2.1.3 Participants and settings

Recruitment was conducted from September 2022 to March 2023 through advertising the study on social media platforms (Instagram, Facebook advertisements, LinkedIn, Telegram), dissemination of electronic study flyers through corporate E-mails (National University Health System (NUHS) and Agency for Science, Technology and Research (A*STAR)), by word of mouth (among family and friends), advertising the study on the CrowdTaskSG online portal initiated by the Singapore government for engaging the citizens, and through collaborating with polyclinics in the West region of Singapore. Interested individuals contacted the study team via phone or E-mail. Recruitment was also conducted on-site in polyclinics located at the West region of Singapore to identify potential women. A two-step screening procedure was adopted. First, women who expressed interest in participating in this study were pre-screened for eligibility via phone. Second, women who met the study criteria (except diabetes status) and provided informed consent were invited to attend a diabetes screening conducted at the study site (Human Development Research Centre (HDRC), National University of Singapore).

### 2.2 Study procedures

#### 2.2.1 Screening procedures

Women were reminded to fast for at least 10 hours before attending the screening visit. Two diabetes screening tests, i.e. a glycated hemoglobin (HbA_1c_) finger prick test followed by a 75g Oral Glucose Tolerance Test (OGTT), were conducted in succession to determine women’s diabetes status before enrolling them to the study. At the screening visit, women first underwent a HbA_1c_ finger prick test (DCA Vantage^®^ Analyzer, Siemens) and provided basic information about their demographics and general health. Women who demonstrated HbA_1c_ ≥ 6.5% at screening visit were excluded from the study and advised to consult their medical doctors. Each woman who had HbA_1c_ < 6.5% was then given an oral glucose solution containing 75g of sugar. After 120 minutes, blood sample was drawn again. The collected blood samples were analysed to measure fasting 0-min plasma glucose (FPG) and 120-min or 2-hour plasma glucose (2hPG) concentrations. Women who had non-diabetic glucose concentrations determined by the OGTT results, i.e. FPG < 7.0 mmol/L and 2hPG < 11.1 mmol/L, were finally included in the study. Otherwise, women were excluded from the study and advised to consult their medical doctors.

#### 2.2.2 Baseline procedures

Demographics were self-reported and baseline anthropometric measures (body weight and height) were recorded. Women were also requested to self-report their general physical and mental health and well-being using self-administered questionnaires (as described in Section 2.6). At baseline visit, after an overnight fasting of at least 10 hours, blood samples were drawn for additional biochemical analyses (i.e, HbA1c, full blood count, insulin, blood markers of inflammation (hsCRP), lipid and liver function tests).

#### 2.2.3 Wearable devices

A continuous glucose monitoring system (CGMS) wearable device (FreeStyle Libre Pro iQ, Abbott Laboratories Limited) was applied to monitor individuals’ subcutaneous glycemic variability for 14 days and women were blinded to the CGMS readings. Each woman was also given an Oura Ring (Ōura Health Ltd.) paired with the Oura proprietary mobile App to collect continuous lifestyle data (physical activity, sleep duration, bedtime heart rate and heart rate variability) throughout the study period (as described in Section 2.6).

### 2.3 Study groups

#### 2.3.1 Randomization

Randomization was performed after baseline assessments and procedures. The randomization strategy (block size of 4) was developed independently by a statistician. Eligible women were randomized to either the Control group or Intervention group based on a 1:1 allocation ratio.

#### 2.3.2 Control group

Women randomized to the Control group received a standard mHealth package that comprised:

1. an Oura Ring paired with Oura mobile App:
  to monitor PA, sleep and bedtime heart rate throughout study period
2. an electronic diary (e-Diary):
  to log dietary information and report perceived stress level (if any) over 14 days from baseline

#### 2.3.3 Intervention group

Women randomized to the Intervention group received an mHealth package that comprised:

1. the standard mHealth package received by the Control group, and
2. a purpose-built study mobile App:
  i. to upload photos of food and beverages for review by a research dietitian,
  ii. to self-report perceived stress level on a 7-point Likert scale, and
  iii. to receive 2 sessions of personalized, messaging-based lifestyle recommendations delivered by a team of health coaches through a virtual chatroom

### 2.4 Intervention

#### 2.4.1 Review of lifestyle behavior

A team of health coaches from multi-disciplinary domains (diet, PA, nursing and public health) reviewed women’s lifestyle data (dietary intake, step count, time spent being sedentary, sleep duration, self-reported stress level). Women were encouraged to wear the Oura Rings and upload photos of food and beverages if lifestyle data was deemed insufficient for evaluation by the health coaches. Health coaching lasted for 4 weeks per woman, and personalized recommendations were delivered via the study mobile App once every 2 weeks to address the lifestyle needs of the individuals.

#### 2.4.2 Personalized lifestyle recommendations

Lifestyle recommendations were guided by the information available on the publicly accessible website of the Singapore Health Promotion Board (14) and the HealthHub website (15). Personalized recommendations were delivered by the health coaches if any of the following conditions was met: (1) the women did not follow the My Healthy Plate recommendations (Singapore Health Promotion Board My Healthy Plate), their diets lacked a decent portion size of healthy food (i.e. fruits, vegetables, protein, wholegrain, less than 2 servings of fish per week) or were high in refined carbohydrates, saturated fat or added sugar (based on the research dietitian’s review of women’s dietary information), (2) weekly average of steps count was below 7,000 steps per day (based on Oura Ring data), (3) weekly average of sleep score was below 80 (based on Oura Ring data), and (4) weekly stress levels were deemed extreme that require intervention by the health coaches (based on women’s self-rating).

#### 2.4.3 Virtual incentives

Virtual incentives in the form of congratulatory messages (e.g. “keep up the good work!”) were delivered to the women who made improvements over time as well as those who maintained satisfactory performance and did not require health recommendations.

### 2.5 Primary outcomes

#### 2.5.1 Feasibility

Feasibility measures included the access to participants, rate of recruitment and retention, proportion of women who met the inclusion criteria, use of mHealth packages, completion of assessments at baseline and endpoint visits, adherence to study duration and procedures, and data completeness. For the Intervention group, we additionally examined the availability of photos of food and beverages needed for dietary assessments, and the feasibility of delivering personalized recommendations by the health coach using the virtual chatroom offered by the study mobile App.

#### 2.5.2 Preliminary efficacy

The preliminary efficacy of the pilot RCT was determined by comparing between the Intervention and Control groups on the mean change from baseline in clinical health and well-being outcomes associated with T2D, i.e. FPG, 2hPG following a 75g of oral glucose load, body weight, blood pressures, lipid profile, perceived general well-being and mental well-being as determined by: (1) depression: based on the Beck’s Depression Inventory (BDI)-II measures (16)), and (2) anxiety: based on the State-Trait Anxiety Inventory (STAI) measures (17).

### 2.6 Explanatory variables

#### 2.6.1 Baseline characteristics

Baseline data included age, marital status, occupation, highest education level, monthly household income, household members, time since the first diagnosis of GDM, number of pregnancies affected by GDM, medical history, history of cigarettes smoking and alcohol drinking and past experience in using mobile Apps and wearable devices for self-monitoring of lifestyle behaviours.

#### 2.6.2 Anthropometrics

Body weight (kg) was measured without shoes using a calibrated digital scale (SECA GmbH & Co. KG). Two measures were recorded to the nearest 0.1 kg, and a third measure was obtained if the first and the second measures differed by > 0.2 kg. The average of the measures was reported.

Body height (cm) was measured without shoes using an automatic weighing scale (SECA GmbH & Co. KG). Two measures were recorded to the nearest 0.1 cm, and a third measure was obtained if the first and the second measures differed by > 1.0 cm. The average of the measures was reported.

Body Mass Index (BMI) was calculated as body weight (in kilograms) divided by height (in meters squared) and categorized based on the Asian cut-off points (underweight: < 18.5 kg/m^2^; normal weight: 18.5-22.9 kg/m^2^; overweight: 23.0-24.9 kg/m^2^; pre-obese: 25.0-29.9 kg/m^2^, and obese: ≥ 30.0 kg/m^2^) (18). Body fat mass (kg) and lean body mass (kg) were assessed using a bioimpedance analysis instrument (SFB7, ImpediMed Ltd.).

#### 2.6.3 Blood pressures

Women were asked to rest for 5 minutes, then systolic blood pressure and diastolic blood pressure were measured twice using an automated digital monitor (HBP 1320, Omron Corp.). A third measure was obtained if the first and the second measures differed by > 10 mmHg. The mean of the measures was reported.

#### 2.6.4 Biomarkers

Blood samples were collected following a minimum 10 hours of overnight fast by a trained phlebotomist using a standardized procedure, and the biochemistry analyses were conducted by the National University Hospital Referral Laboratories. In this study, the following blood biomarkers were analyzed and reported: HbA_1c_ (% and mmol/L), FPG and 2hPG concentrations following a 75g of oral glucose load (mmol/L), Triglycerides (mmol/L), LDL (low-density lipoprotein) cholesterol (mmol/L) and HDL (high-density lipoprotein) cholesterol (mmol/L).

#### 2.6.5 Dietary intake

Each woman was requested to self-report their intake of meals including snacks (i.e. photos of food and beverages, date, time, main ingredients of their food and beverages, place, home-cook vs dine out, companion) via the e-Diary for 14 days from baseline.

During the intervention period, women randomized to the Intervention group were additionally requested to continue uploading photos of food and beverages to the study mobile App for the research dietitian to review the women’s dietary intake and deliver personalized dietary recommendations through the virtual chatroom.

#### 2.6.6 Physical activity, sleep duration and bedtime heart rate

The Oura Ring (Ōura Health Ltd) is a novel wearable that detects body movement, sleep or wake states using multiple sensors (accelerometer, temperature). The Oura Ring has been validated against medical grade actigraphy and polysomnography (19–21). Each ring was paired with Oura’s proprietary mobile App to provide continuous and objectively measured lifestyle data, i.e. step counts, time spent on intensity-based physical activity, time spent being sedentary, sleep duration, bedtime heart rate and heart rate variability. Each woman was encouraged to wear her Oura Ring throughout the study period.

During the intervention period, Oura Ring data contributed by the Intervention group were reviewed by the health coaches to offer lifestyle recommendations through the virtual chatroom. Recommendations were personalized and tailored to the needs of the women.

#### 2.6.7 Perceived stress

Each woman was requested to self-report any stressful events (if any) that they encountered during the first 14 days of the study. They were asked to share information about the date and time of the reported stressful events, reasons for feeling stressful, and rate their stressfulness on a 7-point Likert scale.

During the intervention period, women randomized to the Intervention group were additionally prompted to rate their stress level via the virtual chatroom for review by the health coaches.

#### 2.6.8 Perceived well-being

A 36-item Short Form Survey (22) that consists of a set of generic, coherent, and easily self-administered questionnaire was used to measure health-related quality-of-life. A Subjective Happiness Scale (23) which is a simple 4-item tool was self-administered to assess an individual’s global subjective happiness.

### 2.7 Data analyses

#### 2.7.1 Data processing

The Oura Ring data were collected on a daily basis. In the context of this study, “Baseline Oura Ring data” was defined as data collected within the first 14 days from the date women made in-person baseline visit to the study site. Data for step count and time spent being sedentary were summarized after removing data points when daily step counts were < 1,800 steps/day (i.e. the lowest 5th percentile) and when women were not contributing data for more than 870 minutes (i.e. the largest 5th percentile). Sleep duration and bedtime heart rate were summarized based on data captured during women’s bedtime (night time) when long sleep periods (≥ 3 hours) as well as intermittent short sleep periods (between 15 mins and 3 hours) were recognized by the Oura Rings. Periods of sleep and naps during day time were not considered in this study.

The photos of food and beverages and other description about meal intake (ingredients, place, companion) submitted by the Intervention group were analyzed by a research dietitian. Similarly, “Baseline diet data” referred to dietary information collected within the first 14 days from the date of baseline visit. Subsequently, the Intervention group continued sharing information about food and beverages throughout the 4-week intervention period. The quality of diet was assessed by the research dietitian to determine whether or not women’s diet was meeting the My Healthy Plate recommendations, whether women were consuming sufficient intake of vegetables, fruits, wholegrain, protein and fish, consuming food or beverages which were high in added sugar, choosing low fat option and avoiding high intake of saturated fat in their diets.

#### 2.7.2 Statistical analysis

Frequencies (percentages), means (standard deviations) or medians (interquartile ranges) were used to summarize the data (questionnaire, surveys, laboratory biochemistry analysis, Oura Ring and study mobile App) collected from women who completed both baseline and endpoint assessments. Statistical tests were performed, e.g. Chi-square or Fisher’s exact test for categorical variables, and two independent sample t-tests or ANOVA for continuous variables. Nonparametric tests were used as appropriate.

Preliminary efficacy was examined by comparing the differences between the two study groups on the change in the interested clinical health and well-being outcomes between study baseline and endpoint. Additional analyses were conducted to only focus on 3 main ethnic groups in the Singaporean population (i.e. Chinese, Malay and Indian) to identify factors associated with the interested clinical outcomes at the end of the study (i.e. glucose concentrations following 75g OGTT, body weight and mental well-being). Associations were assessed using multivariate linear regressions adjusted for potential confounders. A sensitivity analysis was performed to determine if there was any significant discrepancy in the direction and magnitude of associations by including all completers in the multivariate regressions model. Further, multivariable logistic regression models with adjustment for potential confounders were conducted to explore factors associated with prediabetes which was determined by the 75g OGTT values between 7.8 and 11.0 mmol/L. A p-value of <0.05 was considered statistically significant. All statistical analyses were conducted using STATA SE version 18 for Windows (StataCorp LLC). Since this was a pilot study, a sample size calculation was not performed. A minimum pragmatic target of 30 women for each study group was set prior to the study.

### 2.8 Ethical approval

Ethical approval was granted by the National Healthcare Group (NHG) Domain Specific Review Board (DSRB) (Ref: 2021/00843). The design, conduct and reporting of the pilot RCT were in accordance with the Consolidated Standards of Reporting Trials (CONSORT) guidelines.

## 3 Results

**Figure 1:**
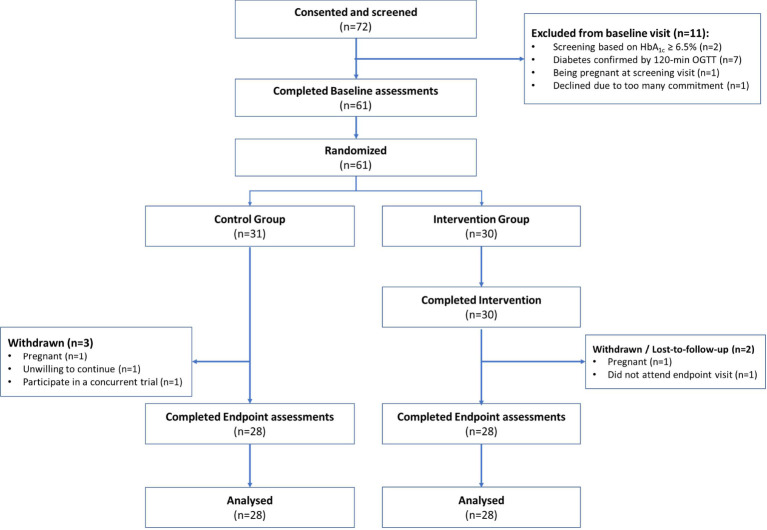
CONSORT diagram.

In total, 196 women from the community expressed interest in this study. 72 (36%) consented for eligibility assessments. **Figure 1** summarizes the flow of participants through the stages of the pilot RCT. Outreach was most successful through advertising the study via NUHS and A*STAR corporate E-mails (38.9%), followed by advertising by word of mouth among family members, friends and relatives (29.2%), social media platforms such as Instagram, Telegram, Facebook advertisement and LinkedIn (25%), CrowdTaskSG online portal (5.6%) and polyclinic in the West region of Singapore (1%). Of the 72 women who attended screening, 61 (84%) were eligible and completed baseline assessments. All of them were randomized at baseline to the Control (n=31) or Intervention (n=30) group, respectively. 56 (92%) completed the study and provided data for analyses. 5 (8%) did not complete the study and the reasons for withdrawal or dropout were self-reported pregnancy (n=2), participating in a concurrent trial (n=1), unwilling to continue (n=1) and did not attend endpoint study visit (n=1).

**Figure 2:**
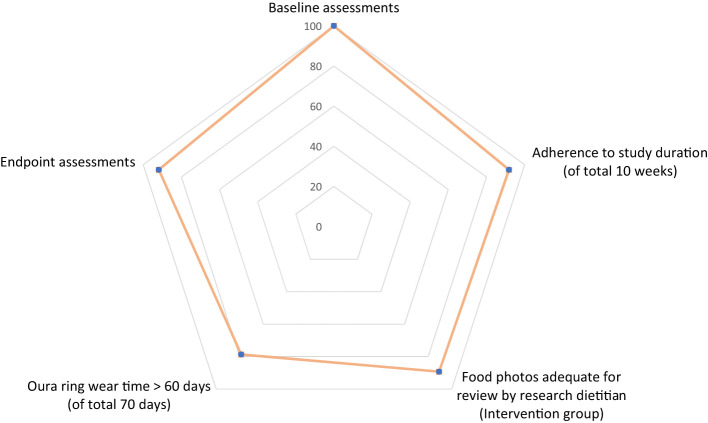
Percentage of women (among 61 eligible women) contributed to various dimensions of the study.

**Figure 2** provides an indication of women’s participation and contribution across various dimensions of the study. Of the 61 women included in the study, all of them (100%) completed baseline assessments, 56 (92%) remain engaged in the study and completed endpoint assessments. Of the 56 completers, 55 (98%) contributed sufficient device data for the subsequent analyses of step count, sedentary time, sleep duration and bedtime heart rate variability, and 48 (85%) actively used the Oura Ring for at least 60 days (of the total 70 days of study period).

For the Intervention group, additional information about dietary intake (photos of food and beverages) were collected in addition to Oura Ring data. 25/28 (89%) women in the Intervention group submitted photos of food and beverages that were deemed adequate for the research dietitian to make assessments and deliver dietary recommendations through the App. Holistic lifestyle data (diet, physical activity, sleep and heart rate) were made available for the health coach to review, make informed feedback and recommendations.

### 3.1 Baseline characteristics

**Tables 1A**, **1B** describe the demographics, clinical characteristics, perceived well-being and lifestyle behaviours of the women randomized to the Control and Intervention groups at baseline. Women in the study (Intervention vs. Control) had mean age (36.3 ± 3.7 vs. 35.4 ± 3.7 years), predominantly Chinese (82% vs. 89%), had their first GDM diagnosed at least 2 years ago (82% vs. 71%) and less than 20% had two GDM-affected pregnancies (18% vs. 14%). Majority of them received tertiary education or above (89% vs. 82%) and had household income of more than USD 7,570 per month (54% vs. 61%).

**Table 1A:** Characteristics of subjects at baseline.

	Control *(n=28)*	Intervention *(n=28)*
*Estimates are presented as n (%), mean ± SD or median (25^th^ - 75^th^ percentile)*		
Demographics		
**Age**, years	35.4 ± 3.7	36.3 ± 3.7
Ethnic group		
Chinese	25 (89.3)	23 (82.1)
Non-Chinese^1^	3 (10.7)	5 (17.9)
Time since first GDM diagnosis		
< 2 years	8 (28.6)	5 (17.8)
2 - 5 years	11 (39.3)	15 (53.6)
>5 - 10 years	9 (32.1)	8 (28.6)
Number of GDM-affected pregnancy		
One pregnancy	24 (85.7)	23 (82.1)
Two pregnancies	4 (14.3)	5 (17.9)
Highest education level		
Secondary	1 (3.6)	n/a
GCE A Level / Polytechnic / Diploma	4 (14.3)	3 (10.7)
Degree	15 (53.6)	16 (57.1)
Post-graduate	8 (28.6)	9 (32.1)
Household income per month, USD equivalent		
< 5,160	6 (21.4)	3 (10.7)
5,160 - 7,570	3 (10.7)	6 (21.4)
7,570 - 10,851	11 (39.3)	9 (32.1)
> 10,851	6 (21.4)	6 (21.4)
Prefer not to answer	2 (7.1)	4 (14.3)
Clinical characteristics		
**Body mass index^2^**, kg/m^2^	22.7 ± 4.0	24.0 ± 4.4
**75g Oral Glucose Tolerance Test**		
0-min plasma glucose concentration, mmol/L	4.9 ± 0.4	4.7 ± 0.4
120-min plasma glucose concentration, mmol/L	7.1 ± 1.6	6.8 ± 1.4
**HbA_1c_,** mmol/mol	34.8 ± 3.1	33.9 ± 2.7^3^
**Systolic blood pressure**, mmHg	108.8 ± 7.9	114.4 ± 10.2
**Diastolic blood pressure**, mmHg	71.4 ± 6.0	74.5 ± 8.5
**Bedtime heart rate**, beat per minute	62.9 (59.0 – 68.1)	62.3 (57.8 – 67.2)
**Bedtime heart rate variability**, milliseconds	44.0 (34.0 – 56.0)	43.0 (34.0 – 58.0)
**Lipid profile**		
HDL-C cholesterol, mmol/L	1.4 ± 0.2	1.4 ± 0.3
LDL-C cholesterol, mmol/L	3.1 ± 0.7	3.1 ± 0.6
Triglycerides, mmol/L	0.9 ± 0.4	0.9 ± 0.6
**Mental wellbeing**		
BDI-II score	5.5 (3.5 – 9.0)	6.5 (2.0 – 12.5)
STAI-Trait score	36.3 ± 8.9	37.3 ± 9.8
STAI-State score	32.1 ± 9.8	33.5 ± 12.0

SD, standard deviation; GDM, gestational diabetes mellitus; n/a, not applicable; USD, US Dollars (1 USD = 1.35 Singapore Dollars); HbA_1c_, glycated hemoglobin; HDL-C, high-density lipoprotein cholesterol; LDL-C, low-density lipoprotein cholesterol; BDI, Beck’s Depression Inventory; STAI, State-Trait Anxiety Inventory. ^1^ Malay (n=3), Indian (n=4), Burmese (n=1), ^2^ BMI cut-off for Asian, ^3^ data was not available for a subject due to error in sample preparation.

**Table 1B:** Characteristics of subjects at baseline (continued).

	Control *(n=28)*	Intervention *(n=28)*
*Estimates are presented as mean ± SD or median (25^th^ - 75^th^ percentile)*		
Perceived well-being		
**36-item Short Form (scores)**		
Limitations in physical activities	90.4 ± 13.5	92.7 ± 9.5
Role limitation due to physical problem	85.7 ± 28.4	88.4 ± 23.1
Role limitation due to emotional problem	72.6 ± 38.6	77.4 ± 35.2
Limitation in social activities	83.9 ± 17.0	81.3 ± 22.4
Bodily pain	85.3 ± 13.1	85.1 ± 18.0
Emotional wellbeing	77.9 ± 11.7	76.3 ± 16.5
Energy/fatigue (vitality)	51.6 ± 14.7	50.9 ± 18.7
General health perceptions	62.5 ± 14.4	65.0 ± 19.0
**Perceived happiness**		
Subjective Happiness score	4.6 ± 0.6	4.5 ± 0.8
Lifestyle behavior		
**Steps count**, median steps / day	9,583 (8,123 - 11,569)	10,046 (8,092 - 11,813)
**Time spent being sedentary**, hours / day	8.7 ± 1.1	8.1 ± 1.4
**Sleep duration**, hours / day	6.3 (5.4 – 7.2)	6.6 (5.7 – 7.4)
**Never smoked cigarettes**	27 (96.4)	27 (96.4)
**Never consumed alcohol**	13 (46.4)	16 (57.1)
**Dietary intake^4^**		
Meeting MyHealthyPlate^5^ recommendation	*Dietary assessment and recommendation were not applicable*	15 (53.6)
Insufficient intake of fibers (fruits, vegetables)		24 (85.7)
High intake of saturated fat		23 (82.1)
High intake of sweetened snacks or beverages		16 (57.1)

SD, standard deviation; ^4^ applicable to women in Intervention group, ^5^ Singapore Health Promotion Board’s recommendation on healthy eating based on “My healthy plate”.

Majority of the clinical characteristics of the women were within healthy ranges. A small number of the women in both groups were found to have prediabetes (18% vs. 32%), slightly elevated systolic blood pressures (14% vs. 29%), LDL-C > 3.3 mmol/L (39% vs. 29%) and Triglycerides > 1.7 mmol/L (4% vs. 7%). The women’s mental well-being was generally satisfactory with only one woman demonstrating a borderline moderate depression (BDI-II score of 30) and a small number of them indicating probable high-state anxiety: STAI-State score ≥ 46 (7% vs. 14%) or STAI-Trait score of ≥ 52 (7% vs. 14%).

The perceived well-being of the women in both groups were largely similar across the examined domains and no significant difference between the two groups was observed at baseline (**Table 1B**). In terms of baseline lifestyle behaviour, women in the Intervention group (vs. Control) achieved median 10,046 (vs. 9,583) steps per day, spent 8.1 (vs. 8.7) hours per day being sedentary and had mean sleep duration of 6.6 (vs. 6.3) hours per day. Almost half of the women in the Intervention group (vs. 46% in the Control group) had never consumed alcoholic drinks. 96% of the women in this study had never smoked cigarettes. Women randomized to the Intervention group were requested to share photos of food and beverages through the study App and had their baseline dietary intake assessed by the research dietitian. Of 28 women, 53% met the My Healthy Plate recommendations, 75% had decent amount of protein intake, 53% had at least 2 servings of fish per week, and only a quarter of them had decent portion sizes of fruits, vegetables and wholegrain in their diet. While 4% of the women had high consumption of refined carbohydrates, 82% and 57% consumed food that were high in saturated fat and added sugar, respectively.

### 3.2 Preliminary efficacy of intervention

**Figure 3:**
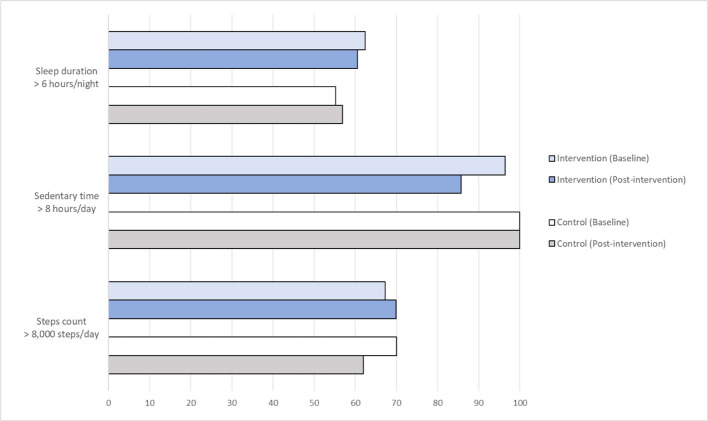
Percentage of women (among 56 completers) and their PA and sleep duration at Baseline and Post-intervention.

**Figure 4:**
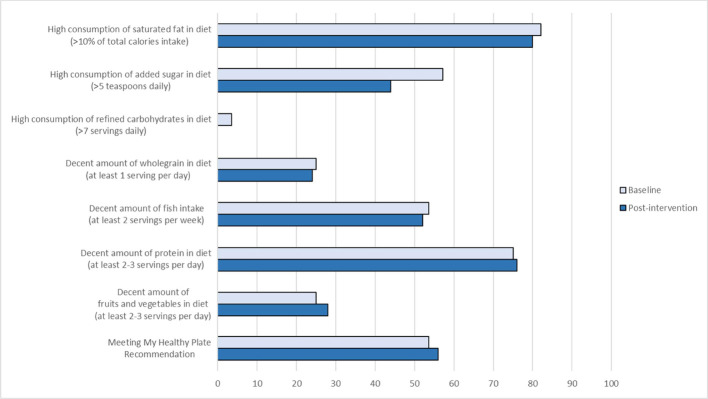
Percentage of women in the Intervention Group (among 25 women) and their dietary intake at Baseline and Post-intervention.

**Figures 3**, **4** summarize the changes in lifestyle behaviors from baseline to post-intervention. Among the 56 completers, a higher proportion of women in the Intervention group achieved daily step counts of at least 8,000 steps per day and reduced sedentary time (or inactive time) as classified by the Oura Ring, but no noticeable improvement was observed for sleep duration (**Figure 3**). Among 25 women who provided adequate number of photos for dietary assessments, none of them demonstrated high consumption of refined carbohydrates after the intervention (**Figure 4**). Slight improvements were observed as women added protein, fruits and vegetables to their diets and reduced the consumption of saturated fat. More noticeable change was observed as the women reduced the consumption of added sugar in their food after the dietary intervention. However, the intake of wholegrain and fish remained inadequate. **Table 2** presents the mean change of clinical health and well-being outcomes from baseline to the end of the study and the observed differences between the study groups were not statistically significant.

**Table 2:** Mean change in preliminary efficacy health and wellbeing outcomes between Intervention and Control groups.

	Change from Baseline mean (95% CI)				Difference between groups mean (95% CI) *p*			
Health and well-being outcomes	Control *n=28*		Intervention *n=28*		Intervention vs. Control			
**Body weight**, kg	0.44	(-0.08, 0.95)	-0.03	(-0.57, 0.50)	-0.47	(-1.19, 0.26)	0.201	
**Body Mass Index^1^**, kg/m^2^	0.15	(-0.07, 0.37)	-0.03	(-0.23, 0.16)	-0.18	(-0.47, 0.11)	0.212	
**75g Oral Glucose Tolerance Test**								
0-min plasma glucose concentration, mmol/L	-0.04	(-0.17, 0.09)	0.14	(-0.01, 0.29)	0.18	(-0.01, 0.37)	0.068	
120-min plasma glucose concentration, mmol/L	-0.36	(-1.10, 0.37)	0.01	(-0.64, 0.66)	0.38	(-0.58, 1.33)	0.435	
**Systolic blood pressure**, mmHg	-1.5	(-4.31, 1.31)	-0.5	(-3.37, 2.37)	1.0	(-2.93, 4.93)	0.612	
**Diastolic blood pressure**, mmHg	-0.25	(-2.52, 2.02)	0.68	(-1.29, 2.65)	0.93	(-2.01, 3.86)	0.528	
Lipid profile								
HDL cholesterol mmol/L	0.0	(-0.07, 0.07)	-0.02	(-0.09, 0.04)	-0.02	(-0.11, 0.07)	0.644	
LDL cholesterol mmol/L	0.01	(-0.15, 0.18)	0.14	(-0.04, 0.31)	0.13	(-0.11, 0.36)	0.287	
Triglycerides mmol/L	0.06	(-0.04, 0.16)	0.01	(-0.16, 0.18)	-0.05	(-0.24, 0.14)	0.691	
Mental well-being								
BDI-II score	-2.39	(-4.36, -0.43)	-2.39	(-5.36, 0.58)	0.0	(-3.49, 3.49)	0.867	
STAI-Trait score	-1.5	(-3.81, 0.81)	-1.30	(-3.91, 1.27)	0.18	(-3.21, 3.57)	0.916	
STAI-State score	-1.89	(-5.23, 1.45)	-1.75	(-6.04, 2.54)	0.14	(-5.18, 5.47)	0.977	
Perceived well-being								
**36-item Short Form (scores)**								
Limitations in physical activities	3.93	(-0.47, 8.32)	-0.54	(-4.60, 3.53)	-4.46	(-10.31, 1.39)	0.115	
Role limitation due to physical problem	6.25	(-5.73, 18.23)	-4.46	(-16.28, 7.35)	-10.71	(-27.16, 5.73)	0.128	
Role limitation due to emotional problem	20.24	(5.17, 35.30)	5.95	(-11.66, 23.56)	-14.29	(-36.94, 8.37)	0.412	
Limitation in social activities	2.23	(-4.50, 8.97)	2.68	(-6.11, 11.46)	0.40	(-10.39, 11.28)	0.916	
Bodily pain	1.96	(-4.29, 8.22)	-0.63	(-8.62, 7.37)	-2.59	(-12.52, 7.35)	0.317	
Emotional wellbeing	2.86	(-1.01, 6.72)	-0.29	(-5.23, 4.65)	-3.14	(-9.28, 2.99)	0.405	
Energy/fatigue (vitality)	2.14	(-4.02, 8.31)	4.11	(-3.19, 11.40)	1.96	(-7.37, 11.30)	0.570	
General health perceptions	2.32	(-1.57, 6.22)	-2.68	(-7.59, 2.23)	-5.0	(-11.13, 1.13)	0.079	
**Perceived happiness**								
Subjective Happiness score	0.05	(-0.12, 0.23)	-0.02	(-0.26, 0.23)	-0.07	(-0.37, 0.22)	0.627	

CI, confidence interval; p, P-value; GDM, gestational diabetes mellitus.

BDI, Beck’s Depression Inventory; STAI, State-Trait Anxiety Inventory.

^1^ WHO BMI cut-off for Asian population.

### 3.3 Factors associated with clinical outcomes

#### 3.3.1 Plasma glucose concentrations

Women with higher BMI had higher 0-min plasma glucose concentrations (or FPG) when compared to women with lower BMI (**Table 3**), however, this positive relationship did not exist for the 120-min plasma glucose concentrations (or 2hPG). Instead, being in the Malay ethnic group was associated with higher 120-min plasma glucose concentrations when compared to their Chinese counterpart. Similar positive relationship was observed in women who had their first GDM diagnosed between 2-5 years ago when compared to women who had their GDM first diagnosed in less than 2 years.

**Table 3:** Additional analysis – results of multivariate regression analysis of health outcomes.

	75g Oral Glucose Tolerance Test								Body weight			
	0-min plasma glucose				120-min plasma glucose							
*Determinants*	*Coeff.*	*95% CI*	*p*	*Std. coeff*	*Coeff.*	*95% CI*	*p*	*Std. coeff.*	*Coeff.*	*95% CI*	*p*	*Std. coeff.*
Group												
Control	*Reference*											
Intervention	-0.01	(-0.22, 0.20)	0.911	-0.02	0.17	(-0.71, 1.04)	0.702	0.06	-3.03	(-6.96, 0.90)	0.126	-0.15
Ethnic group												
Chinese	*Reference*											
Malay	0.47	(-0.10, 1.04)	0.101	0.31	2.65	(0.32, 4.98)	**0.027**	**0.41**	5.47	(-4.99, 15.93)	0.295	0.12
Indian	0.00	(-0.39, 0.39)	0.997	0.00	-0.89	(-2.51, 0.73)	0.270	-0.16	2.17	(-5.10, 9.45)	0.547	0.06
Baseline BMI^1^												
< 23.0 kg/m^2^	*Reference*											
23.0 - 24.9 kg/m^2^	0.19	(-0.09, 0.47)	0.168	0.20	0.42	(-0.72, 1.57)	0.458	0.10	12.04	(6.89, 17.20)	**<0.001**	**0.42**
≥ 25.0 kg/m^2^	0.24	(0.00, 0.49)	**0.047**	**0.34**	-0.15	(-1.13, 0.84)	0.765	-0.05	15.78	(11.34, 20.22)	**<0.001**	**0.75**
Time since first GDM diagnosis												
< 2 years	*Reference*											
2-5 years	0.19	(-0.07, 0.45)	0.145	0.28	1.09	(0.02, 2.16)	**0.046**	**0.37**	0.56	(-4.26, 5.37)	0.816	0.03
>5-10 years	0.23	(-0.08, 0.54)	0.133	0.31	0.21	(-1.06, 1.48)	0.739	0.07	-4.73	(-10.44, 0.97)	0.101	-0.22
	Mental well-being											
	Depression (BDI-II)				Anxiety (STAI-State)				Anxiety (STAI-Trait)			
Group												
Control	*Reference*											
Intervention	2.76	(-0.49, 6.01)	0.094	0.24	3.92	(-1.27, 9.10)	0.134	0.21	2.95	(-0.95, 6.85)	0.133	0.18
Ethnic group												
Chinese	*Reference*											
Malay	8.82	(0.16, 17.48)	**0.046**	**0.35**	12.66	(-1.14, 26.45)	0.071	0.31	7.65	(-2.73, 18.03)	0.143	0.21
Indian	-1.13	(-7.16, 4.88)	0.702	-0.05	-5.55	(-15.14, 4.04)	0.247	-0.16	0.59	(-6.63, 7.81)	0.869	0.02
Baseline BMI^1^, kg/m^2^												
< 23.0	*Reference*											
23.0 - 24.9	2.10	(-2.16, 6.37)	0.323	0.13	0.27	(-6.53, 7.07)	0.937	0.01	2.49	(-2.62, 7.61)	0.328	0.11
≥ 25.0	-0.74	(-4.41, 2.94)	0.686	-0.06	-2.28	(-8.14, 3.57)	0.433	-0.12	-0.34	(-4.75, 4.07)	0.876	-0.02
Time since first GDM diagnosis, year												
< 2	*Reference*											
2-5	-0.61	(-4.60, 3.38)	0.756	-0.05	-4.05	(-10.40, 2.30)	0.203	-0.22	-7.45	(-12.23, -2.67)	**0.003**	**-0.45**
>5-10	3.36	(-1.37, 8.08)	0.157	0.27	-1.72	(-9.25, 5.80)	0.644	-0.09	-2.60	(-8.26, 3.07)	0.357	-0.14

Mutually adjusted multivariate regression model with adjustment for demographics (age, number of GDM affected pregnancy, highest education level, monthly household income) and baseline clinical characteristics (systolic blood pressure, plasma glucose, BDI-II and STAI scores). No multicollinearity was detected. R-square ranged between 0.6 and 0.8 and residuals were normally distributed.

Bold values denote statistically significant results.

Coeff, unstandardized coefficient; Std. coeff, standardized coefficient; CI, confidence interval; p, P value.

BMI, body mass index; GDM, gestational diabetes mellitus; BDI, Beck’s Depression Inventory; STAI, State-Trait Anxiety Inventory.

^1^ Baseline BMI classified by WHO cut-off for Asian population.

#### 3.3.2 Body weight

Women who were being overweight and obese at baseline were associated with higher body weight at the end of the study (**Table 3**). A noticeable magnitude of intervention effect on body weight favoring the Intervention group was observed, however, the effect was not statistically significant.

#### 3.3.3 Mental well-being

When compared to Chinese women who constantly maintained mental well-being within the healthy ranges, Malay women had higher BDI-II scores but none of them demonstrated probable clinical depression at the endpoint assessment. The findings further suggest that women with their GDM diagnosed 2-5 years ago had lower anxiety (STAI-Trait) level than women who had their GDM diagnosed more recently in less than 2 years ago.

#### 3.3.4 Prediabetes

**Figure 5:**
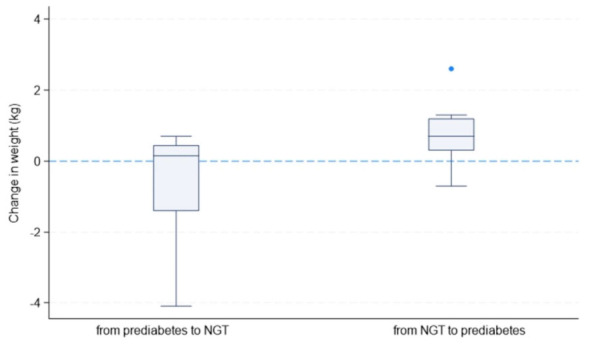
Changes in women’s blood glucose status and body weight between study Baseline and Endpoint (NGT, normal glucose tolerance).

Prediabetes was more common among women who had 2 GDM-affected pregnancies (67%) as compared to women who had only 1 GDM-affected pregnancy (19%). The results of multivariable logistic regression suggest that the odds of prediabetes were significantly associated with higher number of GDM pregnancy after accounting for potential confounders (**Table 4**). **Figure 5** shows the changes in women’s blood glucose status (i.e. normal glucose tolerance (NGT) vs. prediabetes) from study baseline to endpoint in relation to the changes in their body weight between the two timepoints. Initial observations suggested that a small number of the women who had NGT at baseline were found to have prediabetes at the end of the study and this was common among the individuals who gained weight over the two timepoints.

**Table 4:** Additional analysis - factors associated with prediabetes at the end of the study.

			Prediabetes^1^				
*Determinants*		*Unadjusted* *OR*	*95% CI*	*p*	*Adjusted* *OR*	*95% CI*	*p*
Group							
Control		*Reference*			*Reference*		
Intervention		0.83	(0.25, 2.73)	0.763	0.50	(0.10, 2.40)	0.383
Baseline BMI^1^							
< 23.0 kg/m^2^		*Reference*			*Reference*		
23.0 - 24.9 kg/m^2^		2.64	(0.47, 14.89)	0.271	5.31	(0.55, 51.59)	0.150
≥ 25.0 kg/m^2^		2.20	(0.58, 8.31)	0.245	4.50	(0.80, 25.31)	0.088
Time since first GDM diagnosis							
< 2 years		*Reference*			*Reference*		
2-5 years		7.50	(0.84, 66.86)	0.071	7.48	(0.60, 93.26)	0.118
>5-10 years		3.69	(0.36, 37.86)	0.271	1.43	(0.09, 23.82)	0.804
Number of GDM pregnancy							
One pregnancy		*Reference*			*Reference*		
Two pregnancies		**8.44**	**(1.77, 40.38)**	**0.008**	**22.97**	**(2.13, 247.5)**	**0.010**

Multivariable logistic regression model mutually adjusted for covariates, age and ethnic group.

Bold values denote statistically significant results.

No multicollinearity was detected.

OR, odds ratio; CI, confidence interval; p, P value; BMI, body mass index; GDM, gestational diabetes mellitus.

^1^ 120-min plasma glucose concentrations (7.8 - 11.0 mmol/L) determined by 75g OGTT.

^2^ Baseline BMI classified by WHO cut-off for Asian population.

## 4 Discussions

This study identified that the mHealth-enabled holistic approach was acceptable among women with a history of GDM as 91.8% of the 61 enrolled women completed the study, and 78.7% wore the Oura Ring for at least 90% of the study duration. Among the women in the Intervention group, 89.3% provided adequate number of photos of food and beverages for dietary assessments. The collection of holistic lifestyle data from multiple sources was feasible, and the delivery of personalized lifestyle recommendations through the study mobile App was feasible. A more integrated solution could potentially streamline the process and improve the women’s uptake of mHealth strategies.

### 4.1 Recruitment and participant characteristics

Eligible women of this study were mainly recruited through corporate E-mails, among family and friends, and advertising the study on social media platforms. Of the 196 women who expressed interest in this study, 36% were screened and 31% were finally included in the study. Among the women who had a GDM history of 5-10 years, more than 90% were excluded due to T2D and were advised to consult with their doctors for further assessment. Women in the study were predominantly from the Chinese ethnic group, highly educated, had medium-to-high household income and potentially more affluent in relation to the use of social media and technology. The apparent low uptake from the minority ethnic groups and women from low socioeconomic status deserve attention since studies have suggested that economic, socio-cultural and societal factors were associated with risk of GDM and T2D in Asia regions (24, 25). Therefore, improving the recruitment strategy to reach out to these groups of women is warranted.

### 4.2 Preliminary efficacy of lifestyle modifications

Preliminary findings of the present study suggest that the observed difference between groups on the body weight outcome was mainly attributable to weight gain among women with BMI of 25 kg/m^2^ or more in the Control group who did not receive health coaching. Suppressing weight gain could be a practical challenge for women with pre-obesity and obesity in the absence of lifestyle intervention. Therefore, introducing women to lifestyle intervention after GDM might confer clinically important benefits in some individuals although the difference between study groups was not statistically significant (26–28).

In line with the current knowledge, interventions focusing on managing weight or BMI have not been effective in preventing glycemic deterioration after GDM (28). The possible explanation could be that the frequency and intensity of the proposed intervention was not optimal for a subgroup of individuals who are at higher risk of T2D (29). Further, the observed higher odds of prediabetes among women with their first GDM diagnosed 2-5 years ago may be attributable to insufficient or neglected health management as compared to women with more recent GDM diagnosis.

Both study groups demonstrated improvement in mental well-being outcomes at the study endpoint, although the intervention effect was not statistically significant. Preliminary findings of this study suggest that women’s exposure to self-monitoring of physical activity and lifestyle behavior may have improved their mental well-being over time (30). In addition, health concerns related to glucose intolerance are often attributable to physiological and psychological stressors. Sleep deprivation and disturbances increase cortisol levels whereas elevated oxidative stress results in insulin resistance, influencing blood glucose levels which in turn increase an individual’s risk of T2D and other metabolic diseases (31–33). Accordingly, giving attention to reducing anxiety and improving mental well-being may help prevent future risk of T2D.

The incidence rate of T2D after GDM is highly variable across countries and it is relatively higher among Asian populations than among Western populations (34, 35). Furthermore, T2D in Asian populations is characterized by a younger age and lower BMI of onset than in the Western populations (36). Possible explanation for such phenomenon in Asia is that the Asian populations are undergoing rapid socio-economic transformation, creating an environment that can induce obesity and maladaptive inflammatory responses that exacerbate β-cell dysfunction in these regions (37). Singapore is no exception with a GDM prevalence around 23.5% in 2019 (IDF Diabetes Atlas 9th edition Singapore Diabetes Report). The Singaporean GUSTO (Growing Up in Singapore Towards healthy Outcomes) study demonstrated that the risk of developing T2D was 12-fold in Singaporean women 4-6 years after their GDM diagnoses (38).

Asian women could benefit from lifestyle modifications following GDM. Initial improvements in metabolic health and lifestyle behaviors following one year of dietary and physical activity interventions were reported (39). Although group difference was not observed, the incidence of T2D within 3 years postpartum was lowered when Asian women were exposed to diet and exercise interventions (40). Results from observational studies further demonstrated that integrating psychosocial well-being (i.e. offering social support and improving self-efficacy) in lifestyle interventions were positively associated with adopting healthy lifestyle (i.e. increased physical activity, improved diet) and lower stress perception. Similar interventions also reduced BMI and postpartum diabetes status and improved metabolic outcomes (41). The current body of knowledge, taken together, support a holistic approach to improving diet, physical activity and psychosocial well-being for preventing T2D in women with a history of GDM.

### 4.3 Strengths and limitations

The present study demonstrates that it was feasible to identify and recruit women with a history of GDM from the larger community settings. Infrastructures were available to collect holistic lifestyle data including sleep and heart rate variability beyond the conventional focus on diet and PA. Regular feedback offered by the Oura Ring may have increased women’s health awareness and enabled them make actionable goals to increase their daily steps count and reduce time spent on being sedentary. The human-technology interface adopted in the present study was feasible for the health coaches to review individuals’ needs in a holistic manner, and allowed them to tailor their recommendations to improve physical health and mental well-being at an individual level. Recommendations delivered in a personalized and targeted manner showed possibility in improving the intake of fruits and vegetables, and reducing intake of added sugar in their diets. The present study also found initial benefits in reducing the levels of depression and anxiety in the women irrespective of randomization. Although some of these initial findings were encouraging, a larger sample size and sufficient follow-up duration are needed to observe health-related benefits.

Several study limitations deserve to be mentioned. The present study does not have sufficient information about women’s pre-pregnancy risk factors and lacks understanding on the influence of low socio-economic status and minority ethnicity on their risk of dysglycemia and other clinical outcomes. The study mobile App was only made available for Android smartphone users, future studies could aim to extend the outreach to include iOS smartphone users. Due to the short duration of the pilot study, the intervention effects on the clinical outcomes cannot be fully examined. Furthermore, women in the Control group who were also at risk of future T2D were given an opportunity to monitor their lifestyle using the Oura Rings which might have narrowed the observed differences between the two groups.

Taken together, the present study supports adopting a holistic approach to understand women’s lifestyle behaviors including sleep and stress beyond the conventional focus on diet and physical activity. Recommendations can be personalized and tailored to promote overall physical health and mental well-being among women with a history of GDM at an individual level. The proposed holistic approach may also be applicable to preventing GDM that shares largely similar risk factors of T2D. Larger studies with sufficient assessment timepoints and longer follow-up duration are warranted to improve the evaluation of intervention effects on the clinical outcomes. It is also crucial to ensure that the minority ethnic groups and women from lower socioeconomic status are well-represented in future studies. An integrated solution could potentially elevate the efficiency of mHealth strategies and benefit stakeholders involved in holistic lifestyle interventions.

## 5 Conclusions

The holistic approach appeared feasible for developing a holistic understanding of lifestyle behaviors including sleep and stress beyond the conventional focus on diet and PA. Lifestyle recommendations can be personalized and tailored to promote physical and mental well-being among women with a history of GDM. Larger studies with sufficient assessment timepoints, longer follow-up duration and sufficient representation of women from minority ethnic groups and lower socioeconomic status are warranted to improve the evaluation of intervention effects on clinical outcomes.

## Data availability statement

The datasets presented in this article are not readily available because Data preparation and analysis involving other component of the study is ongoing. Requests to access the datasets should be directed to janice.l@nus.edu.sg.

## Ethics statement

The studies involving humans were approved by National Healthcare Group (NHG) Domain Specific Review Board. The studies were conducted in accordance with the local legislation and institutional requirements. The participants provided their written informed consent to participate in this study.

## Author contributions

SL and JE contributed to conception, design of the study and development of the study protocol. YC assessed and evaluated dietary information. MT determined parameters related to women’s metabolic health. SL performed data processing and statistical analysis. SL and CS provided the interpretation of Oura Ring data. SL wrote the first draft of the manuscript and JE provided supervision and reviewed the manuscript. JE obtained the research funding for this study. All authors contributed to the article and approved the submitted version.
